# Comparison of Gadopentetate dimeglumine and Gadobenate dimeglumine in depiction of non-ischemic fibrosis in hypertrophic cardiomyopathy

**DOI:** 10.1186/1532-429X-11-S1-P259

**Published:** 2009-01-28

**Authors:** Andre Rudolph, Hassan Abdel-Aty, Jeanette Schulz-Menger

**Affiliations:** Franz-Volhard-Klinik, Kardiologie, Charité Campus Buch, Universitätsmedizin Berlin, Helios-Klinikum, Berlin, Germany

**Keywords:** Late Gadolinium Enhancement, Hypertrophic Cardiomyopathy, Myocardial Fibrosis, Left Ventricular Remodel, Contrast Administration

## Background

There is a known link between myocardial fibrosis as defined by late gadolinium enhancement (LGE), severity of left ventricular remodeling and ventricular arrhythmias in hypertrophic cardiomyopathy (HCM). Noninvasive quantification of fibrosis may offer the possibility for risk stratification. Due to their often diffuse and blurred character these lesions remain difficult to differentiate from healthy myocardium in several cases.

Recent studies showed the superiority of Gadobenate dimeglumine (Gd-BOPTA) compared with Gadopentetate dimeglumine (Gd-DTPA) in distinguishing infarcted from viable myocardium which can be explained by the higher relaxivity of Gd-BOPTA. Therefore we hypothesized that Gd-BOPTA may have advantages over Gd-DTPA for depiction of non-ischemic fibrosis.

## Methods

We prospectively enrolled eight Patients with clinically established HCM and positive LGE during clinical routine scan with 0.2 mmol/kg Gd-DTPA (0.5 molar). They underwent a second scan at least 72 hours apart from the first CMR exam with 0.2 mmol/kg Gd-BOPTA (0.5 molar) using the same CMR protocol and sequence parameters. None of the patients had renal failure, coronary artery disease or general contraindications for CMR.

LGE was assessed in a short axis stack acquired 15 minutes after contrast administration by using state of the art inversion recovery gradient echo sequence (slicethickness 6 mm, no gap, TE 5.0 ms, FA 30°, matrix 256 × 192) with a TI adjusted to null signal from normal myocardium. Positive LGE was judged positive if signal intensity was above mean + 2 standard deviations of remote myocardium.

Signal intensities of injured myocardium, healthy myocardium, LV cavity and air were measured in identical locations by using anatomical landmarks in dedicated software (CMR42, circle international). Signal to noise ratio (SNR) and contrast to noise ratio (CNR) were calculated.

## Results

We observed no complications relating to contrast administration. Both the SNR of injured myocardium (39.1 ± 15.9 vs. 57.7 ± 25.2, p = 0.02) and the CNR between healthy and injured myocardium (37.2 ± 16.3 vs. 53.0 ± 23.5, p = 0.021) were significantly higher with Gd-BOPTA. SNR of LV cavity was significantly higher (56.9 ± 24.3 vs. 119.0 ± 5.8, p = 0.002) with Gd-BOPTA resulting in a poorer contrast between injured myocardium and blood which did not affect the diagnosis since LGE was exclusively non-subendocardial.

## Conclusion

Gd-BOPTA improves the visualization of non-ischemic fibrosis in HCM. This may offer the potential for a more accurate quantification of focal myocardial fibrosis in this setting.

